# Mental health of medical personnel during the COVID‐19 pandemic

**DOI:** 10.1002/brb3.1881

**Published:** 2020-10-17

**Authors:** Jovana Antonijevic, Iva Binic, Olivera Zikic, Snezana Manojlovic, Suzana Tosic‐Golubovic, Nikola Popovic

**Affiliations:** ^1^ Faculty of Medicine University of Nis Nis Serbia; ^2^ Psychiatry Clinic Clinical Centre Nis Nis Serbia; ^3^ Centre for Mental Health Protection Clinical Centre Nis Nis Serbia; ^4^ School of Electrical Engeneering University of Belgrade Belgrade Serbia

**Keywords:** anxiety, COVID‐19, medical personnel

## Abstract

**Introduction:**

The coronavirus disease 2019 (COVID‐19) pandemic caused significant changes in the everyday functioning of the general population, as well as medical workers. Medical personnel, especially those in direct contact with COVID‐19 patients, could have increased levels of stress, anxiety, and depression. The objective of this study was to explore the mental health status of medical personnel in Serbia during the pandemic by assessing stress levels, symptoms of anxiety, and depression.

**Methods:**

This cross‐sectional study was conducted as an online‐based survey, in the period from 8 April to 14 April 2020, during the COVID‐19 pandemic. The study included 1678 participants, and the snowball sampling technique was used to reach healthcare professionals. The level of stress and symptoms of depression and anxiety were assessed among medical personnel in Serbia by the 10‐item Perceived Stress Scale (PSS), the Beck Depression Inventory IA (BDI‐IA), and the 7‐item Generalized Anxiety Disorder Scale (GAD‐7), respectively.

**Results:**

A total of 1678 participants completed the survey, with a mean age of 40.38 ± 10.32 years, of which 1,315 (78.4%) were women, and 363 (21.6%) were men. Out of these, 684 (40.8%) participants were medical personnel, and 994 (59.2%) were people of other professions. Frontline medical personnel reported higher scores on all measurement tools than second‐line medical personnel (e.g., mean PSS scores: 19.12 ± 5.66 versus 17.53 ± 5.71; *p* = .006; mean GAD‐7 scores: 8.57 ± 6.26 versus 6.73 ± 5.76; *p* = .001; mean BDI‐IA scores: 9.25 ± 8.26 versus 7.36 ± 7.28; *p* = .006). Binary logistic regression showed that the probability of developing more severe anxiety symptoms doubles in frontline medical personnel.

**Conclusion:**

Our findings suggest that frontline medical personnel is under an increased psychological burden during the COVID‐19 pandemic, having higher levels of stress, anxiety, and depression than second‐line medical personnel. Adequate measures should be taken to relieve this burden and preserve the mental health of frontline medical personnel.

## Graphical abstract

The COVID‐19 pandemic put healthcare professionals in challenging situations. This study assessed the impact of working with COVID‐19 patients on parameters of mental health of the healthcare workers. Frontline medical staff with close contact with patients with COVID‐19 reported significantly higher levels of stress, anxiety, and depression than second‐line medical personnel, and they were also found to be twice more likely to develop more severe anxiety symptoms. Measures should be taken to relieve this psychological burden of frontline medical staff.

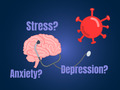

## 1 INTRODUCTION

The recent pandemic of coronavirus disease (COVID‐19), caused by severe acute respiratory syndrome coronavirus 2 (SARS‐CoV‐2), spread rapidly throughout the world after the first reported case in Wuhan, China (Lu et al., 2020). In the Republic of Serbia, the first case of COVID‐19 was confirmed on 6 March 2020. On March 15, the Serbian government declared a nationwide state of emergency and a wide range of counterepidemic measures were progressively adopted (The Government of the Republic of Serbia, 2020).

Increased level of stress is associated with working in health care even in the usual circumstances. Depression, anxiety, sleep disturbances, and burnout were described in that context (Cleary et al., 2018). This pandemic put healthcare professionals in a challenging situation, especially those working in hospitals caring for patients with suspected or confirmed COVID‐19. They were dealing not only with greater infection risk and fear of contagion and spreading the virus to their loved ones (Greenberg et al., 2020; Xiang et al., 2020) but also with emotional disturbances, sleep problems, isolation, lack of contact with their families, extended shifts, and physical exhaustion (Kang et al., 2020).

Previous studies have found that the COVID‐19 pandemic has a severe impact on the mental health of healthcare workers as well as of the general population (Kang et al., 2020; Qiu et al., 2020). According to research on similar outbreaks of severe acute respiratory syndrome (SARS) and Middle East respiratory syndrome (MERS), anxiety and fear were the first symptoms to appear among medical personnel, but depression and post‐traumatic stress symptoms appeared afterward causing severe consequences and had a long‐term effect on the mental health of medical personnel (Chong et al., 2004; Lee et al., 2018).

The World Health Organization defines mental health as, “a state of well‐being in which the individual realizes his or her own abilities, can cope with the normal stresses of life, can work productively and fruitfully, and is able to make a contribution to his or her community” (World Health Organization, 2005). Considering that the most frequent consequences of stressful life events on mental health are the occurrence of anxiety and depression (Fink, 2016), this paper aimed to explore mental health status by examining if medical workers who work with COVID‐19 patients (frontline medical personnel) had higher levels of anxiety, depression, and perceived stress than medical workers who do not work with COVID‐19 patients (second‐line medical personnel).

To our knowledge, our study is the first of its kind conducted in Serbia, and it might be useful in planning and implementing appropriate mental health interventions, support, and follow‐up for the frontline medical personnel.

## 2 METHODS

### 2.1 Study design, participants, and setting

This research was designed as a cross‐sectional study, conducted during the fifth and sixth weeks of the COVID‐19 outbreak in Serbia, and is a part of a larger study. Due to a nationwide lockdown, which was instituted as a counterepidemic measure, the study was conducted via a questionnaire on the online platform 1KA (Centre for Social Informatics, Faculty of Social Sciences, University of Ljubljana, Slovenia).

We used the snowball sampling technique to reach healthcare professionals and the general population. The link to the questionnaire was primarily sent by e‐mail, and each participant was encouraged to disseminate the link further to their contacts, both professional and personal.

### 2.2 Informed consent and anonymity

The survey was anonymous and in accordance with the ethical principles set by the Declaration of Helsinki. Participants were not asked for any information which could be used for identification. The informed consent was located on the introductory page, and participants agreed to it by clicking the “Begin” button. Participation was completely voluntary.

### 2.3 Design of the questionnaire

Sociodemographic questionnaire and questionnaires assessing stress level, anxiety, depression, and resilience were used.

Sociodemographic data were collected on gender, age, marital status, occupation, children, and residence. The occupation was divided into the following categories: frontline doctors, second‐line doctors, frontline nurses, second‐line nurses, and other professions. Since there are no paramedics in Serbia, that answer was not included as an option.

The level of stress was measured by the 10‐item Perceived Stress Scale (PSS), which demonstrated good internal reliability and good construct validity (Cohen, 1988). Respondents were asked to score each of the ten items from 0 (never) to 4 (very often), according to their thoughts and feelings in the previous month. The scores were divided using tertiles, into low, medium, and high stress groups.

The Beck Depression Inventory IA (BDI‐IA) was used for the assessment of depressive symptoms. This scale demonstrated high internal consistency, good test–retest correlation, high concurrent and construct validity, and adequate factorial and discriminant validity (Beck et al., 1988). The inventory consists of 21 groups of four statements, scored on a scale from 0 (normal or absent) to 3 (severe) (Beck & Beamesderfer, 1974). Results were divided into score groups as follows: minimal (<10); mild (10–18); moderate (19–29); and severe depression (30–63) (Beck et al., 1988).

Anxiety symptoms were assessed with the 7‐item Generalized Anxiety Disorder Scale (GAD‐7), which has high reliability, and construct, criterion, factorial, and procedural validity (Hinz et al., 2017; Spitzer et al., 2006). Respondents were asked to rate each item from “not difficult at all” (0 points) to “extremely difficult” (3 points), depending on the influence on their everyday functioning. Final scores were divided into four groups: minimal (0–4), mild (5–9), moderate (10–14), and severe (15–21) anxiety (Hinz et al., 2017; Spitzer et al., 2006).

Brief Resilient Coping Scale (BRCS), consisting of 4 items, was used to assess resilience. This scale demonstrated good construct and criterion validity, good test–retest reliability, and adequate internal consistency (Sinclair & Wallston, 2004). Respondents were asked to rate each item from 1 (“does not describe me at all”) to 5 (“it describes me very well”). The scores were divided using tertiles, into low, medium, and high resilience groups.

One additional question was introduced in the questionnaire. It was a closed‐ended, multiple‐choice question, regarding the dominant thought content related to anxiety and fear. The subjects could choose one or more items as an answer to the question, “What are your fears or anxiety mostly related to?” The answers offered are shown in the results section.

### 2.4 Statistical analysis

Analysis of the collected data was performed in SPSS version 20 (IBM Corp; RRID:SCR_002865). As the Kolmogorov–Smirnov test confirmed that the scores of the used tools were not distributed normally, the nonparametric Mann–Whitney *U* test and Kruskal–Wallis test were used to compare values between groups. Chi‐square test was used to compare the differences in the relative frequency of different score categories, and *Z* test was used for pairwise comparison. We used binary logistic regression analysis to determine the potential impact of working with COVID‐19 patients on the probability of increase in stress, anxiety, and depression levels in medical personnel. Medical workers were classified into two categories for each dimension—0 if they were in the groups with low stress, minimal anxiety, or minimal depression, and 1 if they were in any of the other categories. Three logistic regressions were performed, and the impact factor was working with COVID‐19 patients (frontline or second‐line medical workers), and the dependent variables were indicators of stress, anxiety, and depression. The significance value of 0.05 or less was considered the significance threshold.

Hierarchical regression analysis was performed to determine the possible role of resilience as a mediator between occupation (medical personnel or other professions) and stress, anxiety, and depression. In the first model, a binary variable, occupation, was used as a predictor. In the second model, resilience was also added as a predictor, and we investigated potential changes in the significance of the first predictor variable. The process was repeated three times—for prediction of stress, anxiety, and depression, respectively.

## 3 RESULTS

The entire sample consisted of 1678 participants (1,315 females; 363 males), with the mean age of 40.38 ± 10.32 years. Of this, the group of medical personnel consisted of 684 participants, and the group of other professions consisted of 994 participants. In the group of medical personnel, 177 participants were frontline (75 doctors and 102 nurses) and 507 were second‐line (245 doctors and 262 nurses) personnel (Table 1).

**TABLE 1:** Sociodemographic structure of the sample

			Profession					Total
			Doctor		Nurse		Other professions	
			Frontline	Second‐line	Frontline	Second‐line		
Gender	Male	% (N)	29.3% (22)	24.1% (59)	9.8% (10)	8.0% (21)	25.3% (251)	21.6% (363)
	Female	% (N)	70.7% (53)	75.9% (186)	90.2% (92)	92.0% (241)	74.7% (743)	78.4% (1315)
Marital status	Single	% (N)	25.3% (19)	35.1% (86)	20.6% (21)	12.2% (32)	33.9% (337)	29.5% (495)
	Divorced	% (N)	4.0% (3)	8.2% (20)	10.8% (11)	9.5% (25)	9.9% (98)	9.4% (157)
	Widowed	% (N)	1.3% (1)	1.2% (3)	2.9% (3)	1.9% (5)	1.5% (15)	1.6% (27)
	Married	% (N)	69.3% (52)	55.5% (136)	65.7% (67)	76.3% (200)	54.7% (544)	59.5% (999)
Children	Yes	% (N)	65.3% (49)	56.3% (138)	73.5% (75)	82.8% (217)	55.7% (554)	61.6% (1033)
	No	% (N)	34.7% (26)	43.7% (107)	26.5% (27)	17.2% (45)	44.3% (440)	38.4% (645)
Residence	Urban area	% (N)	97.3% (73)	98.0% (240)	85.3% (87)	82.1% (215)	90.8% (903)	90.5% (1518)
	Rural area	% (N)	2.7% (2)	2.0% (5)	14.7% (15)	17.9% (47)	9.2% (91)	9.5% (160)
Total		% (N)	100.0% (75)	100.0% (245)	100.0% (102)	100.0% (262)	100.0% (994)	100.0% (1678)

Scores of the PSS and BRCS scales were divided into tertiles as follows: for PSS—low (0–15), moderate (16–21), and high (22–40) stress; for BRCS—low (0–13), moderate (14–16), and high (17–20) resilience.

### 3.1 Levels of stress and distribution of stress scores

To investigate levels of stress in these groups, we calculated mean scores and standard deviations. The Cronbach alpha coefficient (Cronbach’s α) for the PSS in our sample was 0.849. The mean score ± standard deviation (*SD*) in the group of healthcare workers was 17.94 ± 5.73, and in people of other professions, it was 18.09 ± 6.27. In the subgroups of medical personnel, mean scores ± *SD* were as follows: frontline doctors, 18.40 ± 5.60; second‐line doctors, 16.26 ± 5.77; frontline nurses, 19.69 ± 5.68; and second‐line nurses, 18.73 ± 5.39.

As shown in Table 2, the chi‐square test was used to analyze the relative frequency of different score groups and significant differences were found (χ^2^ = 12.495, *p* = .014). *Z* test was used for pairwise comparison. The group of frontline medical personnel had a significantly lower percentage of respondents in “low stress” group compared to both second‐line personnel and group of other professions.

**TABLE 2:** Relative frequency of score groups in frontline medical personnel, second‐line medical personnel, and people of other professions, with chi‐square test and *Z* test results

		Frontline medical personnel % (N)	Second‐line medical personnel % (N)	Other professions	Total	Chi‐square
Stress	Low	25.15% (41)^b^	35.29% (168)^a^	35.05% (334)^a^	35.11% (543)	*χ* ^2^ = 12.495, *p* = .014*, *df* = 4
	Moderate	41.72% (68)^a^	40.97% (195)^a^	35.68% (340)^a^	37.88% (603)	
	High	33.13% (54)^a^	23.7% (113)^b^	29.28% (279)^a^	28.02% (446)	
Anxiety	Minimal	28.39% (44)^b^	44.26% (208)^a^	45.34% (418)^a^	43.31% (670)	*χ* ^2^ = 24.831, *p < *.001*, *df* = 6
	Mild	33.55% (52)^a^	28.72% (135)^a^	31.56% (291)^a^	30.9% (478)	
	Moderate	16.13% (25)^a^	14.47% (68)^a^	11.71% (108)^a^	12.99% (201)	
	Severe	21.94% (34)^b^	12.55% (59)^a^	11.39% (105)^a^	12.8% (198)	
Depression	Minimal	68.24% (101)^a^	75.28% (332)^a^	70.32% (623)^a^	71.59% (1056)	*χ* ^2^ = 6.667, *p* = .353, *df* = 6
	Mild	18.24% (27)^a^	16.55% (73)^a^	19.07% (169)^a^	18.24% (269)	
	Moderate	10.14% (15)^a^	5.67% (25)^a^	8.24% (73)^a^	7.66% (113)	
	Severe	3.38% (5)^a^	2.49% (11)^a^	2.37% (21)^a^	2.51% (37)	
Resilience	Low	44.83 % (65)^b^	37.79 % (164)^a,b^	33.52 % (292)^a^	35.93 % (521)	*χ* ^2^ = 13.168, *p* = .010*, *df* = 4
	Moderate	40.00 % (58)^a^	46.08 % (200)^a^	44.20 % (385)^a^	44.34 % (643)	
	High	15.17 % (22)^a,b^	16.13 % (70)^b^	22.27 % (194)^a^	19.72 % (286)	

Note:

Symbols in superscript (^a^ and ^b^) represent the results of the *Z* test; the groups with the same symbol (^a^ and ^a^ or ^b^ and ^b^) do not significantly differ; groups marked with different symbols (^a^ and ^b^) differ significantly. The groups marked with both symbols (^a,b^) do not significantly differ neither from group marked with ^a^ nor from the group marked with ^b^.

* *p *< .05.

### 3.2 Levels of anxiety and distribution of anxiety scores

The Cronbach α for the GAD‐7 in our sample was 0.919. The mean level of anxiety ± *SD* in the group of medical personnel was 7.18 ± 5.94 and in people of other professions 6.34 ± 5.52. Mean levels ± *SD* in the subgroups of medical personnel were as follows: frontline doctors, 7.37 ± 5.68; second‐line doctors, 5.31 ± 4.93; frontline nurses, 9.58 ± 6.57; and second‐line nurses, 8.05 ± 6.16.

Using the chi‐square test, statistically significant differences were found between relative frequencies of score groups (*χ* ^2^ = 24.831, *p* < .001), and pairwise comparison was done using the Z test. Frontline medical personnel had a significantly lower percentage of respondents in the “minimal anxiety” group and a significantly higher percentage of respondents in the “severe anxiety” group compared to both second‐line medical personnel and the group of respondents of other professions.

### 3.3 Levels of depression and distribution of depression scores

For the BDI‐IA, Cronbach’s α in our sample was 0.882. Depression score, expressed as mean ± *SD*, in the group of medical personnel was 7.84 ± 7.57 and in the group of other professions was 8.20 ± 7.68. Mean scores ± *SD* in subgroups of medical personnel were as follows: frontline doctors, 7.73 ± 6.97; second‐line doctors, 6.35 ± 6.45; frontline nurses, 10.65 ± 9.12; and second‐line nurses, 8.34 ± 7.89.

A statistically significant difference was not found between frontline medical personnel, second‐line medical personnel, and respondents of other professions in the relative frequency of score groups, as analyzed by chi‐square test (*χ* ^2^ = 6.667, *p* = .353) (Table 2).

### 3.4 Resilient coping style

In our sample, Cronbach’s α for the BRCS was 0.775. Mean BRCS scores ± *SD* in the groups of our respondents were as follows: 13.81 ± 3.24 in the group of medical personnel and 14.32 ± 3.09 in the group of other professions. In the subgroups of medical personnel, mean scores ± *SD* were as follows: frontline doctors, 14.46 ± 2.92; second‐line doctors, 14.74 ± 2.91; frontline nurses, 12.95 ± 3.28; and second‐line nurses, 12.97 ± 3.35.

Statistically significant differences between relative frequencies of score groups were found using the chi‐square test (*χ* ^2^ = 13.168, *p = *.010). The *Z* test was used for pairwise comparison (Table 2).

### 3.5 Differences between frontline and second‐line medical personnel

As shown in Table 3, statistically significant differences in levels of stress (*p* = .006), anxiety (*p* = .001), and depression (*p* = .006) were found between the frontline and second‐line medical personnel. Frontline medical personnel had significantly higher levels of stress, anxiety, and depression than second‐line medical personnel.

**TABLE 3:** Results of the Mann–Whitney *U* test for determining differences in scores of stress, anxiety, depression, and resilient coping style between frontline and second‐line medical personnel

		N	Mean	Standard deviation	Median	Range	*p*
Stress	Frontline medical personnel	163	19.12	5.66	19.0	31 (6‐37)	.006*
	Second‐line medical personnel	476	17.53	5.71	18.0	33 (0‐33)	
Anxiety	Frontline medical personnel	155	8.57	6.26	7.0	21 (0‐21)	.001*
	Second‐line medical personnel	470	6.73	5.76	5.0	21 (0‐21)	
Depression	Frontline medical personnel	148	9.25	8.26	8.0	39 (0‐39)	.006*
	Second‐line medical personnel	441	7.36	7.28	6.0	45 (0‐45)	
Resilient coping style	Frontline medical personnel	145	13.69	3.19	14.0	16 (4‐20)	.695
	Second‐line medical personnel	434	13.85	3.26	14.0	16 (4‐20)	

* *p *< .05.

Three binary logistic regressions were performed. Our results suggest, as shown in Table 4, that working as frontline personnel is associated with an increase in the probability of exhibiting elevated stress (*p = *.017) and anxiety (*p = *.001) levels, but not depression (*p = *.094) levels, although the significance is close to the 0.05 threshold. In frontline medical personnel, the probability of exhibiting anxiety symptoms in the range of mild, moderate, and severe scores is increased by 100% (exp{B} = 2.003).

**TABLE 4:** Logistic regression—influence of working with patients with COVID‐19 on the probability of exhibiting more severe symptoms of stress, anxiety, and depression in medical personnel

	B	Wald	*df*	*p*	Exp(B)	95% C.I. for EXP(B)	
						Lower	Upper
Stress	0.484	5.613	1	.018*	1.623	1.087	2.423
Anxiety	0.695	11.952	1	.001*	2.003	1.351	2.969
Depression	0.349	2.806	1	.094	1.417	0.942	2.132

* *p *< .05.

### 3.6 Differences between medical personnel and other professions

Medical personnel, frontline and second‐line combined, had significantly higher (*p* = .009) mean levels of anxiety ± *SD* (7.18 ± 5.94) than respondents of other professions (6.34 ± 5.52), as determined by Mann–Whitney *U* test. Also, there was a significant difference in BRCS scores between these two groups (*p* = .005). Respondents of other professions had higher mean BRCS score ± *SD* (14.32 ± 3.09) than medical personnel (13.81 ± 3.24). The differences in stress levels and depression were not found to be statistically significant.

### 3.7 The role of resilience as a mediator between the occupation and stress, anxiety, and depression

Based on the hierarchical regression analysis, the first model, where the only predictor variable was occupation—medical workers or other professions, yielded statistically significant prediction of anxiety (*R* ^2^ = 0.01, *p = *.002; occupation: *β* = 0.08, *p = *.002). After adding resilience as a predictor, the model became significantly better in predicting anxiety (*R* ^2^ = 0.15, *p* < .001; occupation: *β* = 0.05, *p = *.03; resilience: *β* = −0.37, *p < .*001). However, this was only partly at the expense of the significance of the occupation (0.002 vs. 0.03), showing that the influence of the occupation only partly reflects trough resilience, but not enough for the occupation to lose its statistically significant contribution. Hence, resilience is only a partial mediator between occupation and anxiety. Conversely, this was not the case with stress and depression; the one‐variable model did not show significant prediction of stress (*R* ^2^ = 0.00, *p = *.659; occupation: *β* = −0.01, *p = *.659) and depression (*R* ^2^ = 0.00, *p = *.494; occupation: *β* = −0.02, *p = *.494). However, adding resilience to these models significantly improved them, although not at the expense of the significance of occupation (for stress: *R* ^2^ = 0.16, *p < *.001; occupation: *β* = −0.04, *p = *.08; resilience: *β* = −0.40, *p < *.001; for depression: *R* ^2^ = 0.17, *p < *.001; occupation: *β* = −0.05, *p = *.039; resilience: *β* = −0.41, *p < *.001).

### 3.8 Dominant anxiety‐producing thought content

The multiple‐choice question was answered by 1547 respondents, of which 53.8% (*N* = 832) gave only one answer, and 46.2% (*N* = 715) checked two or more answers.

The anxiety‐producing thought content related to infecting families and loved ones with SARS‐CoV‐2 was reported by 70.4% frontline doctors and 70.2% frontline nurses. In contrast, this thought content was present in 35.2% of second‐line doctors and 49.8% of second‐line nurses. In addition, frontline doctors had a significantly lower percentage of thought content “unrelated to the current pandemic” compared to second‐line doctors and both frontline and second‐line nurses (Table 5).

**TABLE 5:** Chi‐square test and *Z* test of dominant anxiety‐producing thought content in doctors and nurses during the COVID‐19 pandemic

	Frontline		Second‐line		Total	*χ* ^2^	*df*	*p*
	Doctors % (N)	Nurses % (N)	Doctors % (N)	Nurses % (N)				
Unrelated to the current pandemic % (N)	19.72% (14)^c^	34.52% (29)^a^	48.46% (110)^b^	37.45% (91)^a^	39.04% (244)	20.577	3	.000*
Related to myself being infected with SARS‐CoV‐2 % (N)	12.68% (9)^a^	19.05% (16)^a^	15.42% (35)^a^	18.93% (46)^a^	16.96% (106)	2.238	3	.525
Related to me infecting my family or people I hold dear with SARS‐CoV‐2 % (N)	70.42% (50)^b^	70.24% (59)^b^	35.24% (80)^c^	49.79% (121)^a^	49.6% (310)	45.349	3	.000*
Related to the possibility of me dying if I get infected with SARS‐CoV‐2 % (N)	5.63% (4)^a^	11.9% (10)^a^	14.1% (32)^a^	10.7% (26)^a^	11.52% (72)	4.065	3	.255
Related to my family or people I hold dear getting infected with SARS‐CoV‐2 % (N)	39.44% (28)^a^	35.71% (30)^a^	39.21% (89)^a^	37.86% (92)^a^	38.24% (239)	0.375	3	.945
Related to the possibility of members of my family or people I hold dear dying if they get infected by SARS‐CoV‐2 % (N)	36.62% (26)^a^	30.95% (26)^a^	29.52% (67)^a^	29.63% (72)^a^	30.56% (191)	1.451	3	.694

Note

Symbols in superscript (a, b and c) represent the results of the *Z* test; the pairs with the same symbol (a and a or b and b or c and c) do not significantly differ; pairs marked with different symbols (e.g., a and b) differ significantly.

Abbreviation:*df*, degree of freedom.

* *p* < .05.

## 4 DISCUSSION

To our knowledge, this is the first mental health investigation in the wake of the COVID‐19 pandemic in Serbia. Our research was done one week before the peak of the number of new confirmed COVID‐19 cases in Serbia, during the fifth and the sixth weeks since the beginning of the outbreak (Coronavirus COVID‐19). The studies conducted during the SARS epidemic showed that psychological responses of medical personnel in epidemics vary significantly depending on the phase of an epidemic. Namely, anxiety is highest and irrational beliefs about the current disease are most frequent in an early stage of an epidemic. As time goes by, anxiety levels drop and the number of irrational beliefs lowers due to gaining knowledge about the nature of the pathogen and the disease itself and having time to adapt to new working conditions (Leung et al., 2005).

Our results showed that levels of stress, anxiety, and depression were significantly higher in frontline than in second‐line medical personnel and that the probability of developing more severe anxiety symptoms doubles in frontline medical personnel. These differences could possibly be explained by the distress caused by unfamiliarity with the pathogen, known characteristics of the disease itself, direct contact with confirmed patients, longer work hours, work burden, exposure to much death and dying, having to make difficult decisions regarding patient treatment (Holmes et al., 2020), and with differences in dominant anxiety‐producing thought content, such as fear of infecting loved ones. It should be taken into account that there is a possibility that these symptoms could persist even after the end of the current pandemic, as it was the case during the SARS and Ebola epidemics (Tam et al., 2004). Delayed onset of post‐traumatic stress disorder may also occur after an acute phase of a pandemic (Mak et al., 2009).

Not only frontline medical workers exhibit higher levels of anxiety, although they are the most affected. All medical personnel differ significantly in the level of anxiety from the group of other professions, which could be explained by the increased risk of working with undiagnosed or asymptomatic COVID‐19 patients.

Our findings show that all medical workers have significantly lower levels of resilience compared to the group of other professions. As a previous study has shown that medical workers with low resilience are more likely to develop anxiety compared to medical workers with high resilience (Mosheva et al., 2020), this could also be a factor contributing to higher anxiety levels in medical personnel.

Since one’s ability to cope with adverse life situations significantly affects investigated mental health outcomes (World Health Organization, 2005), we also examined the role of resilience as a mediator variable between the occupation and levels of stress, anxiety, and depression. It was shown that resilience partially explains the effect of occupation on anxiety, but not on stress and depression. However, all three models are significantly improved when resilience is added as a predictor variable, in addition to the occupation. This result may prove useful to future studies.

There are several limitations to our study. Firstly, given the limited time frame, we used the cross‐sectional design, and conducting a longitudinal study would be necessary to determine a causal link and long‐term effects of the COVID‐19 pandemic on mental health. Secondly, the snowball sampling method was based on the nonrandom selection of the sample and may indicate selection bias. Finally, assessment of mental health was based on an online survey and self‐report measures, which have disadvantages compared with an in‐person interview.

## 5 CONCLUSION

Our study has shown, though with limitations, that levels of anxiety, depression, and stress during the COVID‐19 pandemic are higher in medical personnel working with COVID‐19 patients than in second‐line medical personnel, and that the probability of developing more severe symptoms of anxiety doubles in frontline medical personnel. In addition, it was shown that all medical personnel had higher levels of anxiety and stress than respondents of other professions.

It is the authors’ opinion that, in accordance with our findings, measures should be taken to reduce the psychological burden on the frontline workers in situations such as the COVID‐19 pandemic. Furthermore, it is essential to monitor the mental health of medical personnel even after this pandemic is over in order to make an early diagnosis of any possible long‐term conditions, and provide them with adequate treatment.

## Conflict of interest

The authors have no conflicts of interest to declare.

## AUTHOR CONTRIBUTIONS

*Conceived and designed the research and collected data*: Jovana and Iva

*Equally contributed to the paper and shared first authorship*: Jovana and Iva

*Made significant contribution to the design of the study*: Nikola

*Analyzed and interpreted the data*: Nikola, Jovana, Iva, and Snezana

*Wrote the manuscript*: Jovana, Iva, and Nikola

*Made critical revisions to the manuscript*: Snezana, Olivera, and Suzana

All authors have read and approved the final version of the manuscript and agreed to be accountable for all aspects of the work.

### Peer Review

The peer review history for this article is available at https://publons.com/publon/10.1002/brb3.1881.

## Data Availability

The data that support the findings of this study are available from the corresponding author upon reasonable request.
